# lncRNA FOXD2-AS1 promotes hemangioma progression through the miR-324-3p/PDRG1 pathway

**DOI:** 10.1186/s12935-020-01277-w

**Published:** 2020-05-24

**Authors:** Tiancheng Zhao, Jiayu Zhang, Cong Ye, Leilei Tian, Yezhou Li

**Affiliations:** 1 Department of Endoscopic Center, The Third Hospital of Jilin University, Changchun, 130000 Jilin China; 2 Department of Gastrointestinal Colorectal and Anal Surgery, The Third Hospital of Jilin University, Changchun, 130000 Jilin China; 3 Department of Obstetrics and Gynecology, The Third Hospital of Jilin University, Changchun, 130000 Jilin China; 4 Operating Room, The Third Hospital of Jilin University, Changchun, 130000 Jilin China; 5 Department of Vascular Surgery, The Third Hospital of Jilin University, Changchun, 130000 Jilin China

**Keywords:** FOXD2-AS1, miR-324-3p, PDRG1, Hemangioma, ceRNA

## Abstract

**Background:**

Long non-coding RNAs (lncRNAs) FOXD2 adjacent opposite strand RNA 1 (FOXD2-AS1) are reported could function as tumor promoter in several cancers. However, its role in hemangioma was not reported to yet.

**Methods:**

Expression level of FOXD2-AS1 in hemangioma tissues and cells was explored using quantitative reverse-time PCR. Cell counting kit-8 (CCK-8) assay, colony formation assay, wound-healing assay, and transwell invasion assay were conducted to measure the roles of FOXD2-AS1. In addition, the levels of markers for proliferation and Epithelial-Mesenchymal Transition were investigated. Connection of FOXD2-AS1 and mcroRNA-324-3p (miR-324-3p) or miR-324-3p and p53 and DNA damage regulated 1 (PDRG1) was analyzed with bioinformatic analysis method and dual-luciferase activity reporter assay.

**Results:**

Here, we found that FOXD2-AS1 was highly expressed in proliferating-phase hemangioma tissues compared with the involuting-phase hemangioma tissues. Functionally, FOXD2-AS1 knockdown suppressed cell proliferation, colony formation, migration, and invasion in vitro. Conversely, overexpression of FOXD2-AS1 promoted tumor growth in vitro. Mechanistically, FOXD2-AS1 inversely regulated miR-324-3p abundance in hemangioma cells. We also found FOXD2-AS1 acted as a competing endogenous RNA (ceRNA) by directly sponging miR-324-3p to regulate PDRG1 expression. In addition, the knockdown of PDRG1 reversed the stimulation effects of FOXD2-AS1 overexpression on HA cells.

**Conclusion:**

To conclude, our study sheds novel light on the biological roles of FOXD2-AS1 in hemangioma, which may help the development of targeted therapy method for cancer.

## Background

Hemangioma (HA) is one of the most commonly occurred cancer type in children and the female patients account for about 75–80% of all patients [1]. HA often possess a life cycle that composed by both proliferation and involution stages. HA can be occurred in many regions of human body and the management of HA is highly personalized [2]. As reported, the abnormal proliferation ability of endothelial cells can stimulate the progression of HA [3]. Unfortunately, the understanding of mechanisms behind HA progression is still poor.

Human genome project indicates about 98% of genomic transcripts were non-coding RNAs (ncRNAs), which have been regarded as junk gene for a long time [4]. However, emerging evidence has suggested instead of being junk gene in genome, ncRNAs are found to have crucial roles in development [5]. Long non-coding RNA (lncRNA) is a type of ncRNA at the length of over 200 nucleotides [6]. Importantly, roles of lncRNAs in cancer tumorigenesis have been appreciated in recent years [7]. For instance, Wang et al. [8] identified a lncRNA-TF-mRNA regulatory network that contribute to hepatocellular carcinoma progression. In addition, Zhao et al. [9] revealed that lncRNA was capable to regulate cisplatin-resistant in epithelial ovarian cancer.

FOXD2 adjacent opposite strand RNA 1 (FOXD2-AS1) is a lncRNA that has been reported to be aberrantly expressed in cancers. For example, FOXD2-AS1 was found elevated expression in glioma, and correlated with high WHO grade [10]. Functional assays showed FOXD2-AS1 regulates the proliferation and metastasis of glioma cells via serving as sponge for microRNA-185-5p (miR-185-5p) to affect high mobility group A2 (HMGA2) expression and PI3K/AKT signaling pathway [10]. Moreover, FOXD2-AS1 was upregulated in cisplatin resistance non-small cell lung cancer (NSCLC), and the knockdown of FOXD2-AS1 improved NSCLC cells drug sensitivity by reducing cell growth, cell migration, and cell invasion through miR-185-5p/sine oculis homeobox homolog 1 (SIX1) axis [11]. However, to the best of knowledge, the roles of FOXD2-AS1 in HA remain unknown and therefore is desired to be explored.

In this work, expression level of FOXD2-AS1 in HA cells and normal cell was explored. Effects of FOXD2-AS1 on HA cell proliferation, colony formation, migration, and invasion were investigated using gain-of and loss-of function experiments. Importantly, competitive RNA theory was used to understand the mechanisms underlying FOXD2-AS1 regulated HA cell behaviors.

## Materials and methods

### Clinical samples

16 paraffin-embedded tissues from proliferative hemangiomas and 14 from involuting hemangiomas were collected at The Third Hospital of Jilin University. Informed consent was obtained from patients. Study protocol was approved by Ethic Committee of The Third Hospital of Jilin University.

### Cell lines and cell transfection

HAs-derived endothelial cells (HDEC) and hemangioendothelioma cell line EOMA cells used in this work were incubated in Dulbecco’s Modified Eagle’s medium (DMEM; Thermo Fisher Scientific, Waltham, MA, USA) in supplement with 10% fetal bovine serum (FBS; Thermo Fisher Scientific). Human umbilical vein endothelial cells (HUVECs) were maintained in M200 medium (Thermo Fisher Scientific) contains Low Serum Growth Supplement (LSGS; Thermo Fisher Scientific). All these cells were purchased from Cell Bank of Chinese Academy Sciences (Shanghai, China).

Small interfering RNA against FOXD2-AS1 (si-FOXD2-AS1) or p53 and DNA damage regulated 1 (PDRG1, si-PDRG1) and the corresponding controls (siR-NC) were purchased from RibioBio (Guangzhou, Guangdong, China). miR-324-3p mimic and the corresponding control (miR-NC) were also bought from RiboBio. pcDNA3.1 contains the coding sequence of FOXD2-AS1 named pcFOXD2-AS1 was obtained from GenScript (Nanjing, Jiangsu, China). Cell transfection was conducted using Lipofectamine 2000 (Thermo Fisher Scientific).

### Cell counting kit-8 (CCK-8) assay

CCK-8 assay was used to explore the effects of FOXD2-AS1, miR-324-3p, or PDRG1 on cell viability. Cells with the density of 1 × 10^4^ cells/well were incubated in 96-well plate and incubated at the above-described cell culture condition. At 0, 24, 48 and 72 h after cell seeding, culture medium were removed, cells were washed with PBS, and the add 10 μl CCK-8 reagent (Beyotime, Haimen, Jiangsu, China) to each well and continuously cultured at 37 °C for 2 h according to the manufacturer’s instructions. Optical density at 450 nm was measured using microplate reader (BioTek Instruments, Winooski, VT, USA).

### Colony formation assay

Colony formation assay was used to explore the effects of FOXD2-AS1, miR-324-3p, or PDRG1 on colony formation ability. 1000 cells were plated in 6-well plate and cultured for 14 days. Subsequently, colonies were fixed with paraformaldehyde, stained with crystal violet, and washed with phosphate-buffered saline. Finally, colonies numbers were counted with CKX41 microscope (Olympus Corporation, Tokyo, Japan).

### Wound-healing assay

Wound-healing assay was used to explore the effects of FOXD2-AS1, miR-324-3p, or PDRG1 on cell migration ability. Cells were seeded at a density of 5 × 10^4^ cells/well in 6-well plate and incubated to 95–100% confluence. Then, wound was created at cell surface and washed with PBS. At 24 h of incubation, cell images were captured with CKX41 microscope.

### Transwell invasion assay

Transwell invasion assay was used to explore the effects of FOXD2-AS1, miR-324-3p, or PDRG1 on cell invasion ability. 1 × 10^5^ cells in serum-free medium were seeded to the upper chamber of Matrigel pre-coated insert. Medium contains FBS was filled into lower chamber. After growth for 48 h, invaded cells were fixed with paraformaldehyde. Then, cells were stained by crystal violet and counted with CKX41 microscope.

### Quantitative reverse-time PCR (qRT-PCR)

RNA extracted from cells was isolated with TRIzol reagent (Beyotime) and reverse transcribed into complementary DNA with PrimerScript kit (Takara, Dalian, Liaoning, China). qRT-PCR was performed at ABI 7500 (Applied Biosystems, Foster City, CA, USA) using TB Green Fast qPCR Mix (Takara) with the following primers: FOXD2-AS1, 5ʹ-TGGACCTAGCTGCAGCTCCA-3ʹ (forward) and 5ʹ-AGTTGAAGGTGCACACACTG-3ʹ (reverse); PDRG1, 5ʹ-GAAAAACTGCGGAAGCAACT-3ʹ (forward) and 5ʹ-CCCCATCTTGGTTCTTGAGT-3ʹ (reverse); proliferating cell nuclear antigen (PCNA), 5ʹ-TGATGAGGTCCTTGAGTG-3ʹ (forward) and 5ʹ-GAGTGGTCGTTGTCTTTC-3ʹ (reverse); matrix metalloproteinase-2 (MMP‐2), 5ʹ-TGATTCTGGTCGCTCAGATG-3ʹ (forward) and 5ʹ-CTTGTTTCCCAGGAAGGTGA-3ʹ (reverse); matrix metalloproteinase-9 (MMP‐9), 5ʹ-GGACCATGGGGATCCTTAC-3ʹ (forward) and 5ʹ-AACACAAGGCTGCCCATTAC-3ʹ (reverse); E‐cadherin, 5ʹ-TAACCGATCAGAATGAC-3ʹ (forward) and 5ʹ-TTTGTCAGGGAGCTCAGGAT-3ʹ (reverse); N‐cadherin, 5ʹ-CAACTTGCCAGAAAACTCCAGG-3ʹ (forward) and 5ʹ-ATGAAACCGGGCTATCTGCTC-3ʹ (reverse); GAPDH, 5ʹ-TATGATGATATCAAGAGGGTAGT-3ʹ (forward) and 5ʹ-TGTATCCAAACTCATTGTCATAC-3ʹ (reverse); miR-324-3p, 5′-ACTGCCCCAGGTGCTGCTGG-3′ (forward) and 5′-GCGAGCACAGAATTAATACGAC-3′ (reverse); U6 snRNA, 5′-AACGAGACGACGACAGAC-3′ (forward) and 5′-GCAAATTCGTGAAGCGTTCCATA-3′ (reverse). The procedure used was 1 cycle of 95 °C for 5 min, followed by 40 cycles of 95 °C for 10 s, 60 °C for 20 s, and 72 °C for 10 s. Relative gene levels were calculated with 2^−ΔΔCt^ method.

### Bioinformatic analysis

miRNA target for FOXD2-AS1 was predicted with lncBase V2.0, and miR-324-3p was selected for analyses. To explore target for miR-324-3p, TargetScan (http://www.targetscan.org/) was used, and PDRG1 was selected for followingly analyses.

### Luciferase assay

Wild-type and mutant FOXD2-AS1 or PDRG1 sequences were inserted into pmirGLO (Promega, Madison, WI, USA) to obtain wt/mt FOXD2-AS1/PDRG1. HA cells were co-transfected with luciferase vectors and miRNAs using Lipofectamine 2000. Relative luciferase activity was measured after 48 h transfection using dual-luciferase reporter assay system (Promega).

### Statistical analysis

Differences in groups were analyzed with Student’s *t* test or one-way analysis of variance at SPSS version 19.0 (IBM Corporation, Armonk, NY, USA). Data were expressed as mean ± SD. P-value less than 0.05 was regarded as statistically significant.

## Results

### FOXD2-AS1 was upregulated in HA cells

**Fig. 1:**
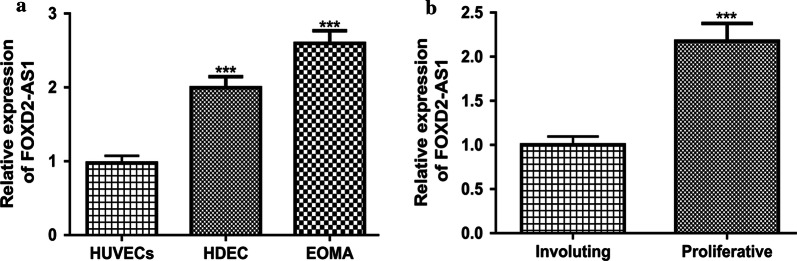
Upregulated FOXD2-AS1 expression in HA. **a** qRT-PCR was used to analyze FOXD2-AS1 expression in HA cells and normal cell. **b** qRT-PCR was used to analyze FOXD2-AS1 expression in proliferative hemangiomas and involuting hemangiomas. FOXD2-AS1: FOXD2 adjacent opposite strand RNA 1; HA: hemangioma; qRT-PCR: quantitative reverse-time PCR

To explore the roles of FOXD2-AS1 in HA, we analyzed its expression in HA cells and normal cell. As indicated in Fig. 1a, FOXD2-AS1 expression was significantly upregulated in HA cells compared with in normal cell. Moreover, we showed FOXD2-AS1 expression level was higher in proliferative hemangiomas than in involuting hemangiomas, indicating FOXD2-AS1 may have a role in contributing HA progression (Fig. 1b).

### FOXD2-AS1 knockdown inhibits HA cell proliferation, colony formation, migration, and invasion

**Fig. 2:**
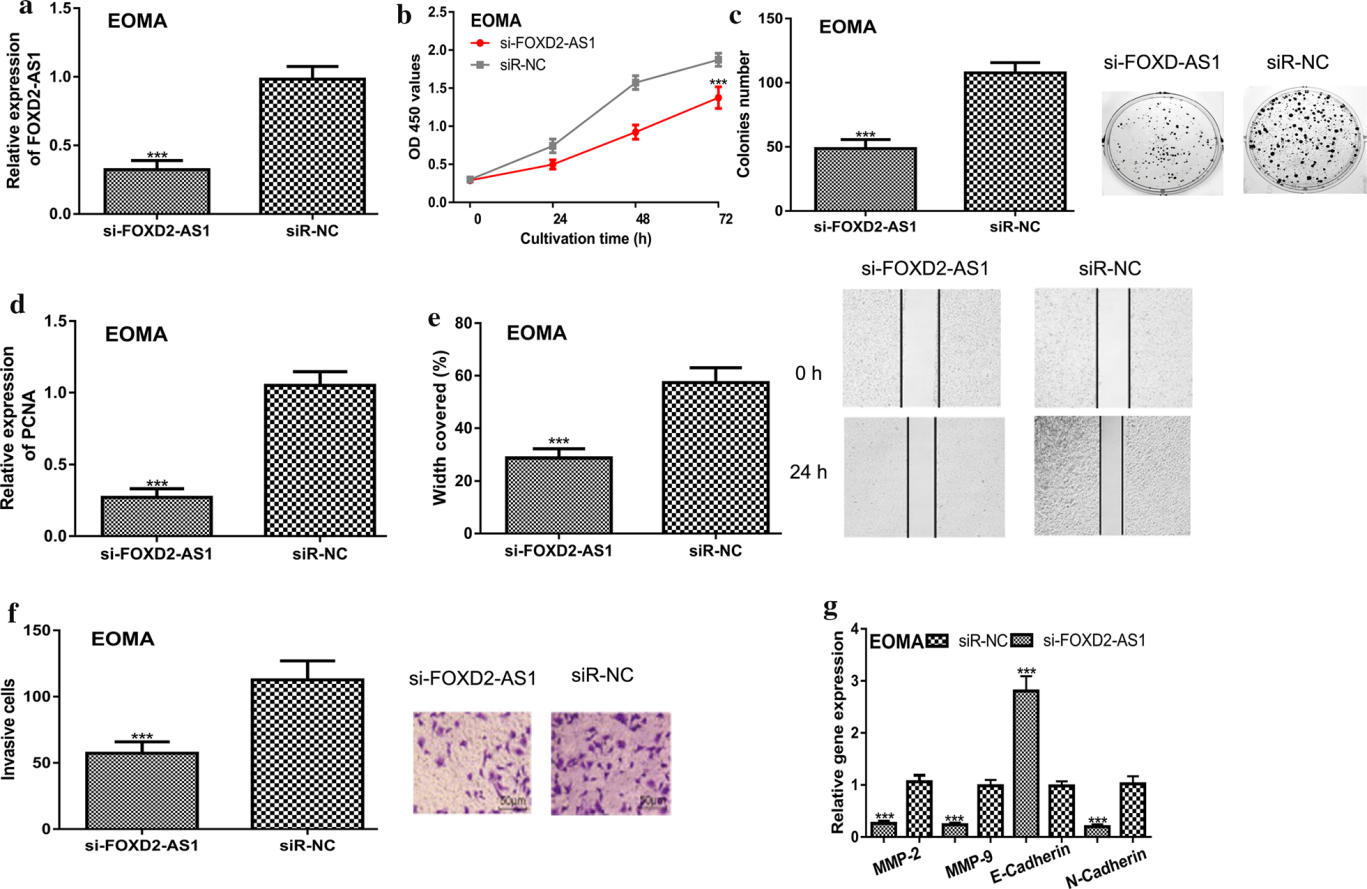
FOXD2-AS1 knockdown inhibits HA cell growth, migration, and invasion. **a** qRT-PCR to detect FOXD2-AS1 expression, **b** CCK-8 assay to detect cell proliferation, **c** Colony formation to detect colony formation ability, **d** qRT-PCR to detect PCNA expression, **e** Wound-healing assay to detect cell migration, **f** Transwell invasion assay to detect cell invasion, and **g** qRT-PCR to detect MMP-2, MMP-9, E-Cadherin, and N-Cadherin expression in HA cell with si-FOXD2-AS1 or siR-NC transfection. FOXD2-AS1: FOXD2 adjacent opposite strand RNA 1; HA: hemangioma; qRT-PCR: quantitative reverse-time PCR; CCK-8: cell counting kit-8; siR-NC: negative control small interfering RNA; si-FOXD2-AS1: small interfering RNA against FOXD2-AS1

To uncover the roles of FOXD2-AS1 in HA, a series of in vitro experiments were conducted. qRT-PCR showed the introduction of si-FOXD2-AS1 significantly decreased FOXD2-AS1 level in HA cell (Fig. 2a). CCK-8 assay and colony formation assay revealed that knockdown of FOXD2-AS1 reduced HA cell growth ability (Fig. 2b, c). We also analyzed the expression of PCNA and found PCNA was decreased in the FOXD2-AS1 knockdown group compared with the normal group (Fig. 2d). In addition, the wound-healing assay and transwell invasion assay revealed that si-FOXD2-AS1 transfection reduced the migration and invasion abilities of HA cells (Fig. 2e, f). Moreover, we showed MMP-2, MMP-9, and N-Cadherin expression was decreased, and the E-Cadherin expression was increased by silencing of FOXD2-AS1 (Fig. 2g).

### FOXD2-AS1 overexpression promotes HA cell proliferation, colony formation, migration, and invasion

**Fig. 3:**
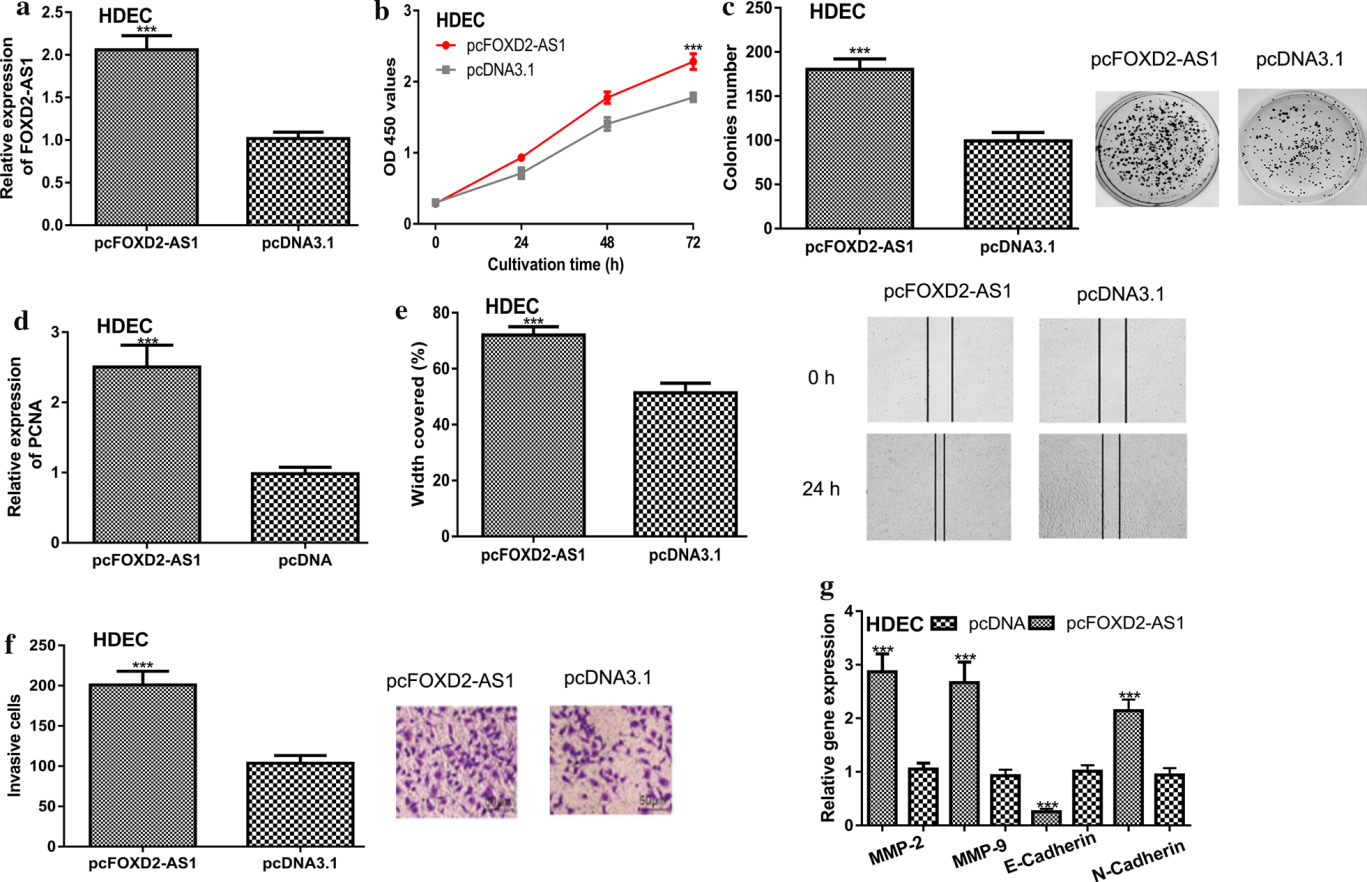
FOXD2-AS1 overexpression promotes HA cell growth, migration, and invasion. **a** qRT-PCR to detect FOXD2-AS1 expression, **b** CCK-8 assay to detect cell proliferation, **c** Colony formation to detect colony formation ability, **d** qRT-PCR to detect PCNA expression, **e** Wound-healing assay to detect cell migration, **f** Transwell invasion assay to detect cell invasion, and **g** qRT-PCR to detect MMP-2, MMP-9, E-Cadherin, and N-Cadherin expression in HA cell with pcFOXD2-AS1 or pcDNA3.1 transfection. FOXD2-AS1: FOXD2 adjacent opposite strand RNA 1; HA: hemangioma; qRT-PCR: quantitative reverse-time PCR; CCK-8: cell counting kit-8

The transfection efficiency of pcFOXD2-AS1 in HA cell was validated by qRT-PCR analysis (Fig. 3a). After pcFOXD2-AS1 transfection, we found cell proliferation and colony formation abilities were significantly stimulated (Fig. 3b, c). As expected, the expression level of PCNA can be elevated by FOXD2-AS1 overexpression (Fig. 3d). Importantly, we showed cell migration and invasion abilities were also significantly upregulated in groups with pcFOXD2-AS1 transfection compared to those with pcDNA (Fig. 3e, f). When FOXD2-AS1 was overexpression, the expression of E‐cadherin was significantly downregulated, while the MMP-2, MMP-9, and N-Cadherin expression was upregulated (Fig. 3g).

We also investigated the roles of FOXD2-AS1 in HUVECs cells by forcing its expression with pcFOXD2-AS1. qRT-PCR confirmed the successful transfection of pcFOXD2-AS1 in HUVECs cells (Additional file 1: Fig. S1a). In addition, functional assays showed the overexpression of FOXD2-AS1 could also increase cell viability (Additional file 1: Fig. S1b–e). Analysis of relative markers showed PCNA, MMP-2, MMP-9, and N-Cadherin levels were increased, while E-Cadherin level was decreased by FOXD2-AS1 overexpression (Fig. 1f).

### miR-324-3p targets both FOXD2-AS1 and PDRG1 in HA

**Fig. 4:**
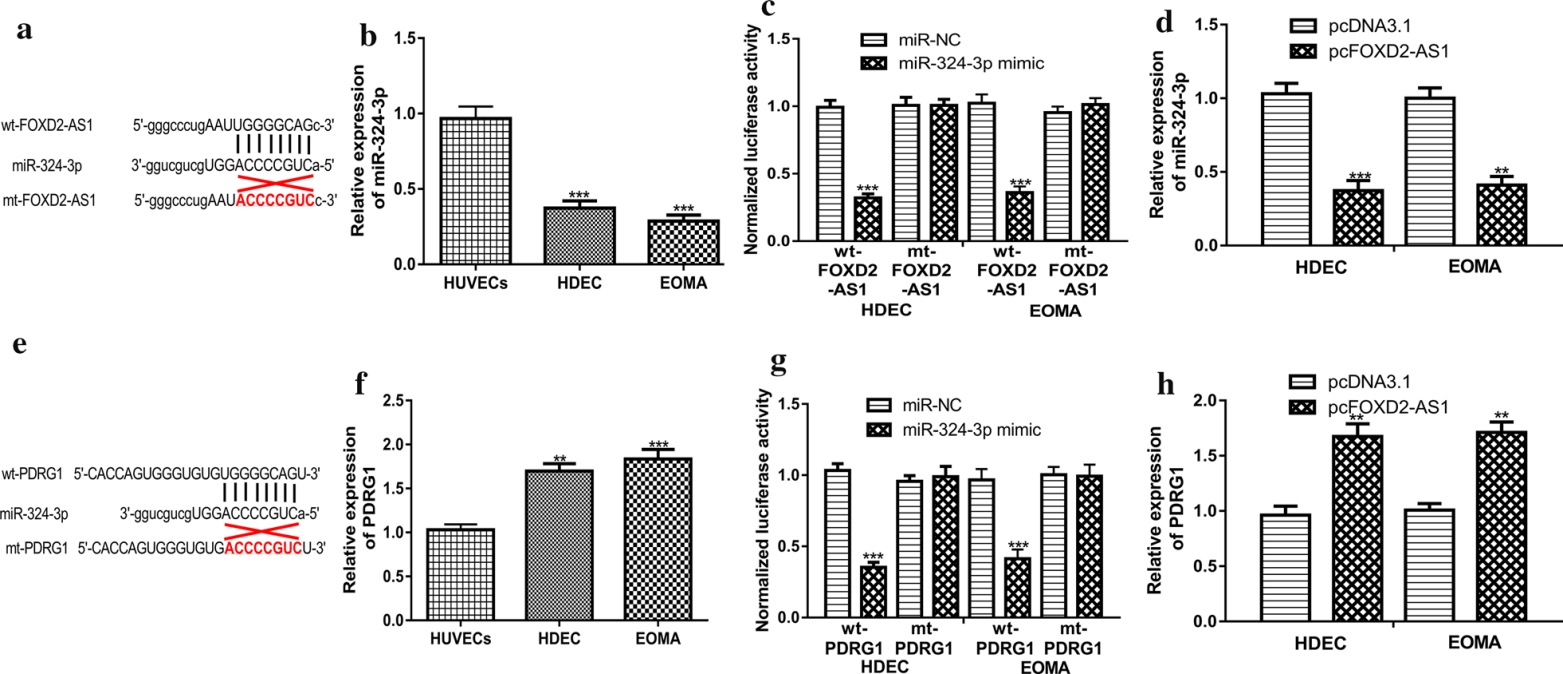
FOXD2-AS1 and PDRG1 shared binding site in miR-324-3p. **a** Putative binding site between FOXD2-AS1 and miR-324-3p. **b** qRT-PCR to detect miR-324-3p in HA cells and normal cell. **c** Relative luciferase activity in HA cells with FOXD2-AS1 luciferase vectors and miRNAs transfection. **d** qRT-PCR to detect miR-324-3p in HA cells with pcFOXD2-AS1 or pcDNA3.1 transfection. **e** Putative binding site between PDRG1 and miR-324-3p. **f** qRT-PCR to detect PDRG1 in HA cells and normal cell. **g** Relative luciferase activity in HA cells with PDRG1 luciferase vectors and miRNAs transfection. **h** qRT-PCR to detect PDRG1 in HA cells with pcFOXD2-AS1 or pcDNA3.1 transfection. FOXD2-AS1: FOXD2 adjacent opposite strand RNA 1; HA: hemangioma; qRT-PCR: quantitative reverse-time PCR; miR-324-3p: microRNA-324-3p; PDRG1: p53 and DNA damage regulated 1; wt: wild type; mt: mutant; miR-NC: negative control miRNA

To explore the molecular mechanism of FOXD2-AS1 in HA, potential miRNA targets of FOXD2-AS1 were predicted and found miR-324-3p was a putative target (Fig. 4a). qRT-PCR results showed miR-324-3p expression was lower in HA cells compared with normal cell (Fig. 4b). Luciferase activity reporter assay showed miR-324-3p mimic introduction decreases luciferase activity of cells with wt-FOXD2-AS1 transfection (Fig. 4c). Moreover, we found overexpression of FOXD2-AS1 could decrease miR-324-3p expression in HA cells (Fig. 4d). Moreover, we analyzed targets for miR-324-3p using TargetScan and found PDRG1 was a putative target (Fig. 4e). We then showed PDRG1 expression was elevated in HA cells in comparison with normal cell (Fig. 4f). Furthermore, the connection of miR-324-3p and the 3′-UTR of PDRG1 was validated by luciferase activity reporter assay (Fig. 4g). qRT-PCR showed overexpression of FOXD2-AS1 could increase the levels of PDRG1 in HA cells (Fig. 4h).

### Silencing of PDRG1 reversed the effects of FOXD2-AS1 on HA

**Fig. 5:**
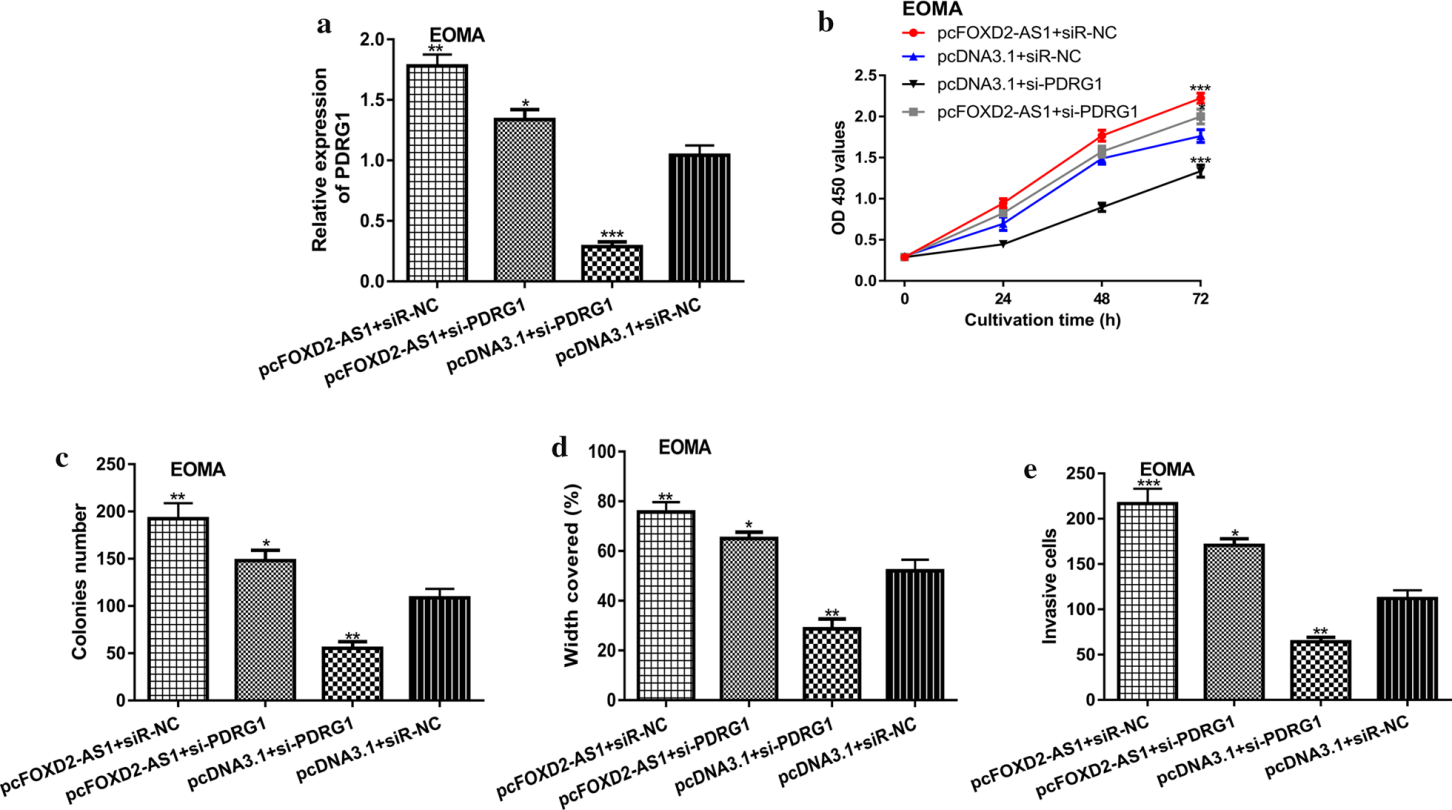
FOXD2-AS1 regulates HA cell behaviors through targeting PDRG1. **a** qRT-PCR to detect FOXD2-AS1 expression, **b** CCK-8 assay to detect cell proliferation, **c** Colony formation to detect colony formation ability, **d** Wound-healing assay to detect cell migration, and **e** Transwell invasion assay to detect cell invasion in HA cell with pcFOXD2-AS1 + siR-NC, pcFOXD2-AS1 + si-PDRG1, pcDNA3.1 + siR-NC, or pcDNA3.1 + si-PDRG1 transfection. FOXD2-AS1: FOXD2 adjacent opposite strand RNA 1; HA: hemangioma; qRT-PCR: quantitative reverse-time PCR; PDRG1: p53 and DNA damage regulated 1; siR-NC: negative control small interfering RNA; si-PDRG1: small interfering RNA against PDRG1; CCK-8: cell counting kit-8

Rescue experiments were performed to examine whether PDRG1 was a functional target for the roles of FOXD2-AS1. We found the si-PDRG1 introduction could decrease PDRG1 and partially reverse the effects of pcFOXD2-AS1 on PDRG1 expression (Fig. 5a). We also showed the knockdown of PDRG1 could inhibit HA cell growth, migration, and invasion (Fig. 5b–e). Importantly, we showed si-PDRG1 could abolished the effects of pcFOXD2-AS1 on HA cell growth, migration, and invasion (Fig. 5b–e).

## Discussions

Previous studies have suggested that lncRNAs have crucial roles in regulating cancer cell proliferation, angiogenesis, migration, and invasion [12]. The oncogenic roles of FOXD2-AS1 in several human cancers have been reported previously but its role in HA remains unknown [10, 11]. In this work, we found FOXD2-AS1 expression level was significantly increased in HA tissues and cells. Through gain-of and loss-of function experiments, we showed that FOXD2-AS1 could promote HA cell proliferation, colony formation, migration, and invasion. We also detected the markers related to proliferation, migration, and invasion. We found FOXD2-AS1 overexpression could stimulate PCNA, MMP-2, MMP-9, N-Cadherin expression, while suppress E-Cadherin expression in HA cells. Importantly, we overexpressed FOXD2-AS1 expression in HUVECs cells and we revealed the overexpression of FOXD2-AS1 was able to stimulate cell malignant behaviors. These results collectively suggested the oncogenic role of FOXD2-AS1 in HA, which is in consistent with the roles of FOXD2-AS1 in other cancer types. Through bioinformatic analysis, luciferase activity reporter assay, qRT-PCR, and rescue experiments, we found FOXD2-AS1 could regulate the malignancy behaviors of HA cells via targeting the miR-324-3p and PDRG1 axis.

miRNAs have been reported to be crucial regulators in cancers. For instance, miR-203 is significantly overexpressed in ER-positive breast cancer and the silence of miR-203 is shown to suppress cytokine signaling 3 expression to regulate cell growth [13]. In addition, a recent study revealed that miR-216-3p was decreased expression in cervical cancer and regulated cell malignant behaviors through inhibiting yes-associated protein signaling through regulating actin-like 6A [14]. Numerous studies have indicated miRNAs are downstream effectors of lncRNAs [15, 16]. miR-185-5p has been indicated as a target for HOXD2-AS1 through two independent experiments [10, 11]. Here, we validated the direct interaction of FOXD2-AS1 and miR-324-3p. miR-324-3p was reported to have dual roles in cancers at a cancer-type basis. For example, Sun et al. [17] revealed miR-324-3p level was elevated in gastric cancer tissues, and its overexpression could promote cancer cell growth, indicating an oncogenic role of miR-324-3p. Besides that, Liu et al. [18] revealed miR-324-3p could suppress nasopharyngeal carcinoma epithelial-mesenchymal transition through targeting WNT2B. Moreover, miR-324-3p was also found could be regulated by lncRNA LINC00261 in cancers [19]. PDRG1 was confirmed as a direct target of miR-324-3p, which is recognized as an oncogene in cancers including gastric cancer and lung cancer [20, 21]. Our work also demonstrated that FOXD2-AS1 regulates HA cell behaviors through regulating miR-324-3p/PDRG1.

Previous studies have suggested several potential strategies for the purpose of cancer treatment in preclinical model [22–27]. For instance, Singh proposed that pre-treatment of cervical cancer with MG132 can facilitate the mitomycin C induced bystander effect and hence to suppress tumor progression [22]. Elevated expression of leptin and resistin was found to regulate the response of cancer cells to chemotherapy [23, 24]. In addition, bitter melon extract was shown to inhibit cancer growth in cancer models, and showed the potential to use it as a potential clinical method for cancer treatment [25–27].

Although we have achieved some progresses by performing these experiments, we have to admit there are limitations in this work. The first is the cases of patients included are relatively small and therefore the conclusions of this work should be validated in large cohorts. Second is we did not explore the roles of FOXD2-AS1 using animal model, which should also be investigated in depth in the future.

## Conclusion

Our results demonstrated that FOXD2-AS1 promotes HA progression by sponging miR-324-3p to regulate PDRG1 expression, indicating lncRNA FOXD2-AS1 may be used as therapeutic targets for HA.

## Supplementary information

**Additional file 1: Fig. S1.** FOXD2-AS1 overexpression promotes HUVECs cell growth, migration, and invasion. **a** qRT-PCR to detect FOXD2-AS1 expression, **b** CCK-8 assay to detect cell proliferation, **c** Colony formation to detect colony formation ability, **d** Wound-healing assay to detect cell migration, **e** Transwell invasion assay to detect cell invasion, and **f** qRT-PCR to detect PCNA, MMP-2, MMP-9, E-Cadherin, and N-Cadherin expression in HA cell with pcFOXD2-AS1 or pcDNA3.1 transfection. FOXD2-AS1: FOXD2 adjacent opposite strand RNA 1; qRT-PCR: quantitative reverse-time PCR; CCK-8: cell counting kit-8

## Data Availability

Data are available upon request.

## Abbreviations

FOXD2-AS1: FOXD2 adjacent opposite strand RNA 1
HA: Hemangioma
qRT-PCR: Quantitative reverse-time PCR
CCK-8: Cell counting kit-8
siR-NC: Negative control small interfering RNA
si-FOXD2-AS1: Small interfering RNA against FOXD2-AS1
miR-324-3p: MicroRNA-324-3p
PDRG1: p53 and DNA damage regulated 1
wt: Wild type
mt: Mutant
miR-NC: Negative control miRNA
si-PDRG1: Small interfering RNA against PDRG1
ncRNAs: Non-coding RNAs
miR-185-5p: MicroRNA-185-5p
NSCLC: Non-small cell lung cancer
HMGA2: High mobility group A2
SIX1: Sine oculis homeobox homolog 1
DMEM: Dulbecco’s Modified Eagle’s medium
LSGS: Low Serum Growth Supplement
PCNA: Proliferating cell nuclear antigen
MMP-2: Matrix metalloproteinase-2
MMP-9: Matrix metalloproteinase-9
