# Supplementary figures and images for: lncRNA FOXD2-AS1 promotes hemangioma progression through the miR-324-3p/PDRG1 pathway
Source: Cancer Cell Int. 2020 May 24;20:189. doi: 10.1186/s12935-020-01277-w (PMC7247140; doi:10.1186/s12935-020-01277-w)

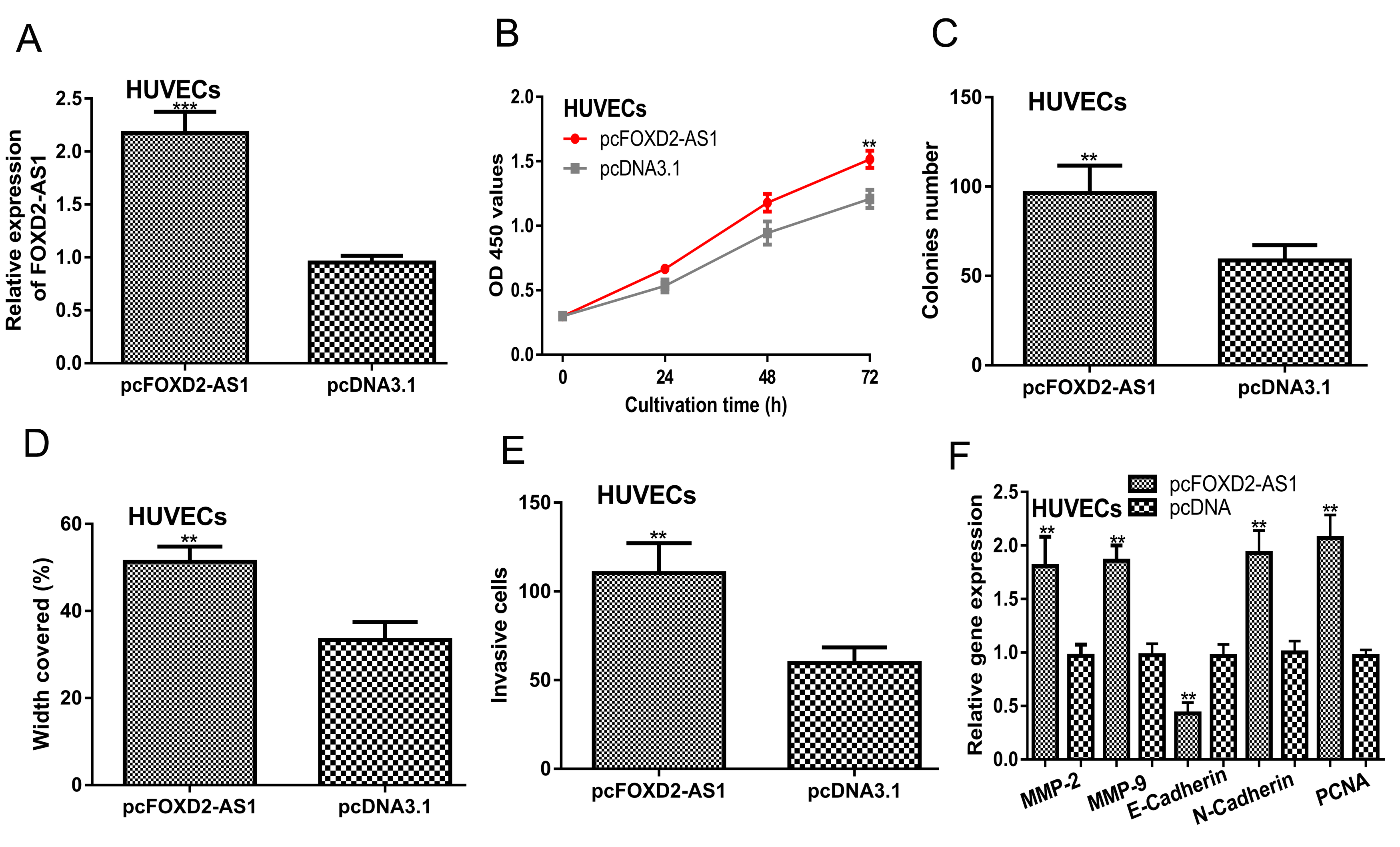

Supplement: Supplementary file 1 — Additional file 1: Fig. S1. FOXD2-AS1 overexpression promotes HUVECs cell growth, migration, and invasion. a qRT-PCR to detect FOXD2-AS1 expression, b CCK-8 assay to detect cell proliferation, c Colony formation to detect colony formation ability, d Wound-healing assay to detect cell migration, e Transwell invasion assay to detect cell invasion, and f qRT-PCR to detect PCNA, MMP-2, MMP-9, E-Cadherin, and N-Cadherin expression in HA cell with pcFOXD2-AS1 or pcDNA3.1 transfection. FOXD2-AS1: FOXD2 adjacent opposite strand RNA 1; qRT-PCR: quantitative reverse-time PCR; CCK-8: cell counting kit-8 [file 12935_2020_1277_MOESM1_ESM.tif]
